# Faecal microbiota transplant revisited - potential metabolic outcomes of regional microbiome mismatches

**DOI:** 10.20517/mrr.2026.29

**Published:** 2026-09-29

**Authors:** Elisabeth A. A. O’Flaherty, Douwe van Sinderen

**Affiliations:** School of Microbiology & APC Microbiome Ireland, University College Cork, Cork T12 K8AF, Ireland.

## INTRODUCTION

Clinical use of faecal microbiota transplant (FMT), from healthy donor stool, is highly effective in treating refractory *Clostridioides difficile* infection, despite safety concerns including bacteraemia and multidrug-resistant organism transmission^[1-3]^. Recent advances in *C. difficile* treatment now include the development of live, stool-derived therapeutics^[4,5]^ and defined, live biotherapeutic products^[6]^. Together, these advances suggest that microbiota-based therapy for *C. difficile* infection is evolving from conventional, donor-derived FMT towards increasingly defined, commercially manufactured microbial therapeutics. This raises the question: what role remains for conventional FMT? The answer may lie beyond *C. difficile*, with therapeutic potential for FMT described in cancer, autism and metabolic disorders^[7-11]^. One area of growing interest is obesity, where current pharmacological therapies, though effective, are limited by cost, tolerability, and weight regain after discontinuation^[12]^. FMT’s therapeutic potential in metabolic disorders is the context in which we revisit the 2025 study by DeLeon *et al*.^[13]^. Here, the authors demonstrated that microbiota transplanted beyond their native intestinal niche persistently reshape regional microbial ecosystems, tissue identity and host metabolism.

## REGIONAL MICROBIOTA MISMATCHING OCCURS ALONGSIDE ALTERATIONS TO THE GUT NICHE

Varied FMT administration routes (oral, endoscopy, colonoscopy, enema) can result in transplanted microbial populations interacting with previously unencountered anatomically and functionally distinct gut niches, in potential conflict with innate microbial ecosystems^[14,15]^. This interaction may disturb a network of specialised spatial ecosystems shaped by local nutrient availability and oxygen gradients, altering recipient intestinal environments following engraftment. In this study, DeLeon *et al.*^[13]^ describe that peroral microbiota transplants (MTs) in antibiotic-treated SPF mice with starting material of either faecal (FMT), caecal (CMT) or jejunal (JMT) origin resulted in engraftment throughout the murine intestinal tract, persisting up to 3 months. Regionally mismatched anaerobic colonisation of the small intestine and engraftment of aerobes in the colon in these post-MT mice raises the question: which conditions permit persistent engraftment outside native niches? Using RNA-seq approaches the authors showed that JMT enriched for a jejunal transcriptional signature in the jejunum and colon of these mice, while FMT enriched for a colonic identity. De Leon *et al.*^[13]^ additionally identified anaerobic colonic bacteria in the human small intestine 1 month post-FMT, accompanied by a subtle enrichment of colonic gene signatures in the duodenum, correlating with interindividual anaerobic colonisation. Treatment of human jejunal enteroids with acellular material from MTs revealed that transcriptional identity was retained following JMT treatment, shifting towards a colonic identity in FMT-treated enteroids. Though these observations from human samples are consistent with the mouse studies carried out by DeLeon *et al.*^[13]^, effects were variable, limited by sample size (*n* = 7), reliant on *in vitro* models, and used duodenal rather than jejunal samples, limiting direct comparison with murine findings and necessitating caution in interpretation. Overall, the data suggest that transplanted microbiota may enhance and induce signatures of their original gut niche, which potentially facilitate their engraftment, persistence and influence on regional tissue function. The concepts of microbiome mismatching introduced by these findings have been highlighted elsewhere^[16,17]^.

## REGIONAL ENGRAFTMENT MISMATCHES POST-MT AFFECT METABOLIC PROFILES IN MICE

DeLeon *et al.*^[13]^ subsequently explored functional impacts related to regional mismatches. Metagenomic sequencing of SPF mouse colonic microbiota post‑transplant showed that CMT had the greatest recovery of microbial pathways and functional potential, perhaps unsurprising as CMT, a proposed intermediate between small and large intestinal microbiota, resulted in the most balanced and widespread engraftment. Metabolic phenotyping by DeLeon *et al.*^[13]^ revealed that JMT mice exhibited metabolic dysregulation including weight gain, low activity and energy expenditure while FMT mice had lower food intake, and CMT mice exhibited the most favourable metabolic phenotype. FMT’s potential impact on mouse metabolism has been reported previously, through transfer of human obesogenic or diabetogenic phenotypes^[18,19]^.

The impact of microbiota transfer on host physiology was further explored by DeLeon *et al*.^[13]^ through transcriptomic liver analyses, revealing signatures enriched for immune pathways in post-FMT SPF mice, while JMT enriched for metabolic and lipid-associated genes, including PPAR signalling and fatty acid metabolism, and CMT recipients again exhibited an intermediate phenotype. These hepatic transcriptional changes are notable in light of evidence implicating PPAR signalling and lipid metabolic pathways in metabolic dysfunction-associated steatotic liver disease (MASLD)^[20]^. As the liver is the first extraintestinal organ exposed to gut-derived metabolites, regional changes in gut microbiota populations could alter the production and delivery of microbial metabolites to the portal circulation, subsequently influencing hepatic programming. Recently, Mi *et al.*^[21]^ reported that oral FMT reduced body weight and steatosis in a MASLD model, providing additional evidence that FMT can modulate metabolic and hepatic phenotypes in mice. Key changes in bioactive microbial metabolites were observed by DeLeon *et al*.^[13]^, including higher levels of the short-chain fatty acid (SCFA) propionate in the small intestine post-FMT compared to JMT, and a reduced proportion of colonic secondary BAs in the JMT group, microbial metabolites with potential hepatic influence^[22,23]^. The observed hepatic transcriptional changes may represent a potential mechanistic link between transplanted microbiota engraftment, metabolite production and whole-body phenotype. Reduced food intake in post-FMT mice compared to JMT mice in this study^[13]^ is consistent with the anorexigenic actions of SCFAs and bile acids, which can influence appetite centrally and through stimulation of gut hormones (e.g., GLP-1 and PYY)^[24-31]^. Notably, changes in colonisation, metabolite levels and liver transcriptomics were mirrored in germ-free experiments (circumventing confounding coprophagic influence) strengthening the microbial transplant effect and potential influence of MT on gut-liver crosstalk. Collectively, these data indicate that MT affects gut microbiota functional potential and metabolites known to influence host metabolism.

Whether these changes would translate clinically from an antibiotic-treated mouse model is not explored here^[13]^. Altered metabolic phenotype following FMT in humans can be interindividual and transient^[8,9,11,32]^ and although FMT has been shown to restore SCFA and BA profiles, this has been in the context of *C. difficile* infection or ulcerative colitis^[33,34]^, requiring exploration in metabolic dysfunction. What is most striking in this study by DeLeon *et al.*^[13]^ is that changes in mouse metabolism are not related to donor phenotype, as this is consistent, but to the regional origin of the transplanted material. Notably, a 2022 study by Zhang *et al.*^[9]^ found that FMT from lean donors led to variable weight loss in obese recipients, with duodenal enrichment of *B. bifidum*, a classically distal anaerobe, observed in responders, supporting a potential association between small intestinal anaerobe engraftment and altered host metabolism. Together, these findings provide insights as to the divergent impacts of regional microbiome mismatches on persistent metabolic programming, highlighting the risks and opportunities associated with the transfer of donor metabolic phenotype.

## CONCLUSIONS AND FUTURE DIRECTIONS

Off-target impacts of FMT are often overlooked, lacking appreciation of regional microbiota heterogeneity and function. In this study, DeLeon *et al.*^[13]^ show that transplanted microbiota engraft throughout the mouse intestine, without appropriately reconstituting regional microbiomes. Clinically, this may call for more anatomically informed microbial formulations, or progression towards compositionally defined microbiome therapeutics^[6]^, particularly as oral encapsulated FMT increases in prevalence, exposing the small intestine to non-native colonic communities. Regional mismatches were accompanied by spatial changes in microbial functional potential, metabolite production, tissue identity and host physiology, suggesting that engraftment mismatches could result in niche engineering, influence inter-organ communication and bias host priorities towards immune or metabolic states. Metabolic shifts observed varied based on regional origin of the transplanted material, with lowest food intake in FMT mice and the most favourable metabolic phenotype in CMT mice. As distal anaerobic communities persisted in the small intestine following CMT and FMT, this raises the possibility that such regional mismatches may eventually be leveraged in novel, microbial-based treatments of metabolic disorders. Previous FMT studies exploring this prospect have focussed on microbiota composition and donor screening in the pursuit of therapeutic efficacy^[8,9,11]^, while here DeLeon *et al*.^[13]^ introduce an additional variable, the regional origin of microbiota. The findings may indicate that recipient response does not depend solely on the transferred microbes, but on whether they are native or naïve to the intestinal niche they engraft. Unpicking these interactions may clarify the heterogenous and transient metabolic changes observed in previous FMT studies, despite metabolically favourable donor selection^[8,9,11]^. At present, limited human data means the clinical significance requires further translation to determine whether beneficial and long-lasting effects would be seen in patients. Additionally, the regulatory frameworks related to expanding FMT beyond *C. difficile* infection must be considered, including rigorous screening, prevention of multidrug-resistant organism transmission and long-term monitoring of recipients. Nevertheless, this study by DeLeon *et al*. raises many important questions and avenues for the future of FMT-based therapeutics^[13]^:

1. As distal anaerobic engraftment of the small intestine may influence host metabolic regulation in mice^[13]^, and humans^[9]^, it would be of interest to:

• explore autologous FMT via the oral or endoscopic route, to determine whether anatomical redistribution of a recipient's own distal microbiota is sufficient to alter host metabolism independent of donor effects, reducing the risk of multidrug-resistant organism transmission.

• functionally characterise whether the distal anaerobes which colonise the small intestine are responsible for improvements in host metabolism, potentially informing future development of defined therapeutic consortia for metabolic disorders.

2. DeLeon *et al.*^[13]^ describe in mice that CMT, an intermediate microbial community, most closely restored engraftment, function and host physiology. Although more invasive than stool collection, clinical protocols for small intestinal fluid collection have been described^[35]^. Clinical investigation of transplanting intermediate microbial populations, or omni-microbial transplants (OMT), is warranted to determine whether these would result in regionally matched microbial communities and more consistent metabolic benefits compared to FMT.

3. While this study focuses on bacterial transplant, recent studies suggest that FMT additionally results in persistent bacteriophage engraftment and that these phages may contribute to post-transplant microbial responses^[36-38]^. Future studies should consider the influence of the intestinal virome alongside bacterial engraftment when interpreting changes following MT.

## DECLARATIONS

### Copyright

© The Author(s) 2026.
